# Oxidative Stress in Aging-Matters of the Heart and Mind

**DOI:** 10.3390/ijms140917897

**Published:** 2013-09-02

**Authors:** Krishnan Venkataraman, Sandhya Khurana, T. C. Tai

**Affiliations:** 1 Department of Gerontology, Huntington University, Sudbury, ON P3E 2C6, Canada; E-Mail: kvenkataraman@huntingtonu.ca; 2 Medical Sciences Division, Northern Ontario School of Medicine, Sudbury, ON P3E 2C6, Canada; E-Mail: sandhya.khurana@nosm.ca; 3 Department of Biology, Department of Chemistry and Biochemistry, Biomolecular Sciences Program, Laurentian University, 935 Ramsey Lake Road, Sudbury, ON P3E 2C6, Canada

**Keywords:** oxidative stress, RONS, cardiovascular, aging, brain

## Abstract

Oxidative damage is considered to be the primary cause of several aging associated disease pathologies. Cumulative oxidative damage tends to be pervasive among cellular macromolecules, impacting proteins, lipids, RNA and DNA of cells. At a systemic level, events subsequent to oxidative damage induce an inflammatory response to sites of oxidative damage, often contributing to additional oxidative stress. At a cellular level, oxidative damage to mitochondria results in acidification of the cytoplasm and release of cytochrome c, causing apoptosis. This review summarizes findings in the literature on oxidative stress and consequent damage on cells and tissues of the cardiovascular system and the central nervous system, with a focus on aging-related diseases that have well-documented evidence of oxidative damage in initiation and/or progression of the disease. The current understanding of the cellular mechanisms with a focus on macromolecular damage, impacted cellular pathways and gross morphological changes associated with oxidative damage is also reviewed. Additionally, the impact of calorific restriction with its profound impact on cardiovascular and neuronal aging is addressed.

## 1. Introduction

The process of aging in the human body is a visible and tangible change that humans will experience in their lifetime. There are several biological models of aging that are accompanied by robust scientific experimental data. Predominantly observed phenomena are damage to DNA, a loss of telomere capping and irreversible oxidative damage in subcellular structures, due to free radical-based oxidation. Oxidative damage in cells can occur due to several reactive molecular species that include reactive oxygen and nitrogen species (RONS), reactive aldehyde species, transition metal intermediates and advanced glycation end (AGE) products [1–4]. A relatively recent review by Sahin and DePinho [5] has a widely accepted model for tissue stem cells that illustrates the interconnections between oxidative stress, DNA damage and gene regulation that signals either cell cycle arrest or apoptosis. The aging of organs and systems in the human body is not equivalent, and it appears that organ systems with greater cell turnover suffered the most accumulated DNA damage [6]. Harman’s Free Radical Theory of Aging is based on the understanding that cumulative oxidative damage to DNA and other cellular components and tissues over time causes aging, disease and death [7]. Oxidative stress and damage are an inevitable consequence of aerobic respiration. Oxidative stress is widely accepted to be a perturbation in the balance of free radicals in a cell and the cell’s ability to cope with this change by means of its antioxidant defenses [3,8–10]. The scope of oxidative damage is vast if all body systems are considered. Neurological degeneration leading to clinical presentation of dementia is present in 5%–7% of global population over the age of 60. Dementia is debilitating, disabling and leads to dependency among older adults. It is estimated that the number of older adults with dementia worldwide is around 40 million. It is estimated that by 2050, the number of older adults will have increased to 1.25 billion, or about 22% of global population, and with it, the epidemic of dementia will continue to grow [11]. The increase in the prevalence of cardiovascular diseases directly correlates with age; cardiovascular disease has been determined to be the primary cause of mortality in older adults and is estimated to amount to 80% in individuals aged 65 and above [12]. An estimated 87 million people above 65 years are predicted to live in the USA by 2030 [12]. In Canada, the number of older adults is estimated to be about 20% of its population by 2021 [13]. Furthermore, epidemiological analysis of the correlation between dementia and heart disease reveal a striking relationship between the two pathological states [14]. Vascular pathology and stroke have been associated with cognitive decline, and peripheral atherosclerosis has been suggested to be a risk factor for the onset of Alzheimer’s disease in the elderly [15]. Thus, the knowledge of the pathophysiology of neurological and cardiovascular complications in the elderly is essential. The primary focus of this review is on oxidative damage in the cardiovascular and neurological systems, the interplay between oxidative stress and coping mechanisms and, finally, the current understanding on the cellular pathways that contribute to what we have come to recognize as aging.

## 2. Oxidative Stress

### 2.1. Sources of Oxidative Stress

Oxidative stress is broadly defined as the redox imbalance between free radicals and cellular antioxidant systems. Reactive oxygen and nitrogen species (RONS) are free radicals predominantly synthesized endogenously. The peroxisomes, lysosomes, ER/microsomes, plasma membrane and cytoplasm, all have oxidases and dehydrogenases and are sources for RONS [8], but the biggest source of endogenous free radicals is the mitochondrion, accounting for most of the free radicals generated in cells [1–3]. Free radicals are formed as a result of electron transport in the mitochondrial membrane to generate ATP. The electrons bind diatomic oxygen molecules to form superoxide O_2_^•−^, which is released either inside the mitochondrial matrix or in the inter-membrane space based on whether the superoxide is released from complex I or complex III of the electron transport chain [16,17]. At complex I, the NADH dehydrogenase reduces NADH and leakage of electrons by partial reduction of FMN from this complex can give rise to ROS. At complex III, cytochrome c is reduced, and ubiquinone is oxidized. The semiquinone anion (Q^−^) is formed as an intermediate during the renewal of coenzyme Q and is capable of transferring electrons to O_2_, subsequently giving rise to O_2_^•−^ [18]. About 2% of diatomic oxygen (O_2_) in the mitochondria ends up as O_2_^•−^ [17]. Alternatively, H_2_O_2_ could also either serve as a precursor in the Haber Weiss reaction or, in the presence of metals, like Fe^2+^, in a Fenton reaction to generate the hydroxyl radical (OH•) [19].

The NOX family of NAD(P)H oxidases is a primary source of ROS generation at the plasma membrane, membranes of endocytic and other cellular compartments, as well as the ER and mitochondrial membranes, of both phagocytic and non-phagocytic cells. NAD(P)H oxidase 1 and 2 (NOX1 and 2) are the two major contributors toward ROS generation, with the p47phox subunit being a critical component of the enzyme complex [20]. Superoxide radicals and nitric oxide (NO) primarily serve to stimulate antioxidant defenses in cells by acting as signaling molecules [16,21]. While O_2_^•−^ is not easily transported across membranes, its conversion into hydrogen peroxide (H_2_O_2_) spontaneously or by the action of superoxide dismutase (SOD) or to peroxynitrite (ONOO^−^), as a result of ubiquitously available NO, allows for free radical translocation across the mitochondrial membranes into the cytosol and other cellular compartments. Both hydrogen peroxide and peroxynitrite are highly reactive with a host of targets in the cell [8]. A table of other species of RONS as downstream products of superoxide and nitric oxide have been summarized by Yu and Chung [21]. This reaction of NO and O_2_^•−^ negates the vasodilatory effect of NO, creating peroxynitrite, a powerful oxidant that is capable of oxidizing endogenous antioxidants, like glutathione, thereby further contributing to an increased oxidative stress. Additionally, ONOO^−^ can interact with tetrahydrobiopterin (BH4), a cofactor for the enzyme endothelial nitric oxide synthase (eNOS), consequently resulting in the uncoupling of eNOS [20]. Further, NO can also undergo autoxidation to form NO_2_^−^ and NO_3_^−^, and these are indicators of increased free radicals and oxidative stress within the body [22]. Xanthine oxidase can also result in the formation of the superoxide anion during the oxidation of xanthine and hypoxanthine and can also give rise to H_2_O_2_, which can then be a precursor in the Haber Weiss or the Fenton reactions to generate ROS [23,24]. Further, monoamine oxidases (MAOs) in the outer mitochondrial membrane are also a significant source of ROS generation within the body.

Additionally, metabolites have been increasingly implicated in oxidative damage in the cell. Notable among these are advanced glycation products (AGEs) and hydroxy alkenals. AGEs are formed as a result of reducing sugars, like glucose, reacting non-enzymatically over several weeks with amino groups on proteins and sometimes lipids and DNA. Several protein targets have been observed, such as collagen, tubulin, myelin, complement and fibrinogen. AGEs have been implicated in several pathologies, including formation of amyloid plaques in the development of Alzheimer’s disease [4]. Hydroxy alkenals are formed as a result of degradation of lipid hydroperoxides. Hydroxy alkenals have a longer half-life than free radicals and can attack targets distant from the site of origin. Targets include protein, DNA and phospholipids. They impact cell signaling as intracellular messengers and influence gene expression [25].

The sources of free radicals in cells are not limited to endogenous products of aerobic respiration as the impact of environmental free radicals in the form of smoke from polluted environments, cigarette smoke, asbestos, coal, diesel, chromium, drugs, like adriamycin and bleomycin, radiation and ozone, among others [26].

### 2.2. Oxidative Damage

Excess ROS have been identified as a major source of cellular damage in biological systems. DNA damage is prominent in the presence of excess ROS, with mitochondrial DNA (mtDNA) being most susceptible to ROS-mediated damage, due to its close proximity with the major source of ROS within the cell. Damage due to oxidative stress in DNA is evidenced as a formation of adducts or single-stranded and double-stranded breaks in nuclear DNA. In addition, there is an increase in the formation of modified bases, such as 8-oxo guanine, 8-oxo-7,8-dihydro- 2′deoxyguanosine (8-oxodG), 2-hydroxy adenine, FAPy-adenine, 8-oxoadenine, 5-hydroxycytosine, cytosine glycol and thymine glycol [3]. For a younger cell, the damage is significantly lower than for an aging cell, and much of the damage is promptly and efficiently repaired by base excision repair pathways, nucleotide excision repair pathways, in the case of a damaged base, or a nucleotide or a single-stranded break, and repair of double-stranded breaks occurs via homologous recombination (HR) or non-homologous end-joining (NHEJ) mechanisms [27]. Damage to RNA is significant, with both mRNA and rRNA being impacted. Up to 50% of the mRNA is oxidized in the frontal cortices of Alzheimer’s disease (AD) patients [28]. Oxidized RNA is a feature of Parkinson’s disease (PD), with the damage in both diseases resulting in increased levels of 8 hydroxy guanine (8 OHG) [29].

Proteins can also be impaired by ROS, both structurally and functionally. Damage to proteins occurs via formation of protein carbonyls in several cellular proteins in their amino acids, lysine, arginine, proline and threonine [2,3]. Common examples are carbonic anhydrase III and plasma fibrinogen. A less common oxidation product is the formation of methionine sulfoxide in proteins, like calmodulin. Oxidative damage appears to be tissue and protein-specific, and overall carbonyl levels have been shown to increase with age [3]. This has been attributed to lower cathepsin levels and decreased proteasomal activity. In addition, crosslinked protein products, like lipofuscin, have been shown to directly inhibit proteasomal activity, thereby setting off a cyclic process of oxidized protein by-product accumulation [30]. Highly oxidized proteins are also not subject to proteasomal degradation and end up accumulating in the cell [31].

Hydroxyl radical can damage cell membrane and diminish cell integrity. ROS can also alter lipid peroxidation and modulate several lipoproteins, such as LDL. Damage to extra cellular lipids is evidenced by the accumulation of oxidized LDL, leading to the formation of foam cells from macrophages, which contribute to the progression of atherosclerosis [8,32]. Oxidation of membrane systems in cells results in altered fluidity, physiology and membrane damage, as seen in neurons, macrophages, platelets and red blood corpuscles [33–36].

### 2.3. Cellular Protection from Oxidative Damage

The damage from ROS is usually minimized by a multitude of enzymes and mechanisms that are protective in nature. Antioxidant defenses are both enzymatic and non-enzymatic. Enzymatic defenses employ catalases, thioredoxin reductase, superoxide dismutases (SODs) and glutathione peroxidase (GPx) [3,8,37]. Antioxidants, such as the lipid-soluble vitamins, D, E and K, protect membranes, and the water-soluble vitamin C neutralizes free radicals in blood, extracellular fluid and the cytosol [10]. Other defenses that allow for free radical neutralization by absorbing the reactive electron involve phenolic compounds, such as tocopherols, carotenes, ascorbate, ubiquinols and flavonoids, or glutathione (GSH), albumin and bilirubin, which are of peptide origin [8,38]. In the mitochondria, mitochondrial manganese containing superoxide dismutase (MnSOD or SOD2) is primarily responsible for converting the superoxide anion to H_2_O_2_, which is then converted to water by the enzyme catalase, largely localized in peroxisomes. In the cytosol, copper-containing CuSOD (SOD1) carries out the same function [39]. Additionally, H_2_O_2_ can also be consumed by GPx to convert reduced glutathione (GSH) to its oxidized form (GSSG). The pools of GSH are replenished by the action of glutathione reductase (GSR) on GSSG [39]. Furthermore, uncoupling proteins located in the inner mitochondrial membrane redirect excess energy toward thermogenesis; these UPCs have been suggested to play a role in regulating ROS production within the mitochondria [40].

## 3. The Aging Cardiovascular System

### 3.1. Age-Related Oxidative Damage in the Cardiovascular System—Structural and Functional Changes

Cardiovascular mortality is the highest cause of death in elderly populations [12]. The effect of aging on the vasculature and the structure and function of the cardiovascular system are diverse, and they all converge toward a progressive failure of the system. Structurally, wall thickening of both tunica intima and tunica media of the large elastic artery results in increased arterial stiffness and lesser dilation [41]. This results in elevated systolic pressure and, together with the ensuing endothelial damage, results in left ventricular (LV) hypertrophy. The hypertrophy ensues because of massive myocyte loss, which are replaced by sarcomeres. Moreover, as a compensatory mechanism to make up for lost myocytes, an increase in the volume of myocytes is observed [41,42]. LV hypertrophy is accompanied by hyperplasia of the myocardial cells; at the interventricular septum, this hypertrophy can lead to the obstruction of outflow from the LV and result in an increased afterload in the ventricular wall [12]. As a consequence of arterial stiffening and increased calcification of the aortic valves, aortic stenosis is also seen in 80% of aging population, altogether leading to deterioration of heart functions. Additionally mitral annular calcification (MAC) or calcification and lipid deposition within the mitral valve is also commonly seen in the elderly and is a risk factor for increased incidences of stroke within this population [43]. There is a significant reduction in the synthesis, as well as the bioavailability of NO (discussed in detail below), which leads to increased vasoconstriction, altered NO-dependent signaling in smooth muscle cells and increased permeability of the endothelium in aged models [41]. Moreover, vascular smooth muscle cells undergo hypertrophy and proliferation and migrate to the subendothelial space, where the extracellular matrix shows signs of calcification, collagen deposits and elastin fragmentation as a function of aging [12]. Functionally cardiac output and heart rate is lower in aging individuals, particularly during exercise [41]. This is correlated with the decrease in β-adrenergic responsiveness that effectuates myocardial contractility [42]. The systolic functions of the LV are intact, whereas the diastolic functions are markedly altered, with LV filling being slow earlier in this phase, but may be compensated by increased atrial contraction in the later diastolic phase [41,42]. Further, myocardial contractility, modulated by Ca^2+^-driven action potential, changes with aging, resulting in a prolonged potential, culminating in increased cytosolic Ca^2+^, caused partially by the slower inactivation of the l-type Ca^2+^ current. This eventually leads to prolonged contraction and hampering of myocardial relaxation, thereby impairing filling of the LV [41].

### 3.2. Age-Related Oxidative Damage in the Cardiovascular System—Molecular Mechanisms

Studies show that aging results in an impairment in the electron transport chain during mitochondrial respiration, primarily because of reduced electron transport by complex I and IV, and could consequently result in leakage of electrons and increased ROS production [44]. Certain key components of complex I and IV are encoded by mtDNA and may be altered quantitatively, because of inhibition of synthesis by ROS signaling or enzyme modification and, thereby, inhibition of enzyme activity by excess ROS [45].

This section summarizes the damage mediated by these free radicals in the aging components of the cardiovascular system.

#### 3.2.1. Endothelial Damage, Alterations in Vessel Wall and Atherosclerosis

The health of the vascular endothelium is preserved as the result of a balanced interplay between endothelium-dependent contracting factors (EDCFs) and endothelium-dependent relaxing factors (EDRFs), as well as endothelium-dependent hyperpolarizing factors (EDHF), such as Endothelin-1 (ET-1) [46]. With aging, this balance is skewed by an increased ROS-mediated reduction in NO, the predominant vasorelaxant in the endothelium, as well as an increase in cyclooxygenase and its dependent vasoconstrictor molecules.

Aging results in the decrease of available NO within the endothelium, with NO being the eminent vasodilator molecule. The reduction in available NO is a corollary of the decrease in NO production, which could occur for a variety of reasons. A scarcity in l-arginine, the substrate for NO production or a decreased level or activity of eNOS, the enzyme that synthesizes NO, could all result in diminished NO within the endothelium. Alternatively, the reduction in NO could also occur as a consequence of the limiting amount of tetrahydrobiopterin (BH4), the cofactor for eNOS, which has been reported to be decreased with aging, likely because it is oxidized by increased ROS, resulting in uncoupling of eNOS [47]. Further, the activity of iNOS, the inducible NOS isoform regulated by proinflammatory cytokines, is increased; however this NOS isoform is proatherogenic. An iNOS-mediated increase of NO generates excess NO, which reacts with superoxide to form peroxynitrite. An increased rate of deterioration of eNOS is also evident upon accumulation of methylated by-products of l-arginine, such as NG-NG’-dimethyl-arginine asymmetric (ADMA), which are endogenous inhibitors of eNOS and can restrict its activity [48]. Moreover, l-arginine deficiency may occur because of increased l-arginase activity, the enzyme that degrades l-arginine and conceivably limits arginine as a substrate for eNOS, thus making l-arginase a potential target for therapeutic intervention in the treatment of vascular diseases [49]. In a study on older rats, it was reported that these animals had higher arginase I activity, decreased eNOS and NO and that arginase I was significantly *S*-nitrosylated, likely by elevated iNOS; inhibition of arginase I reversed the uncoupling of eNOS and improved acetylcholine-derived vasorelaxation [50]. The supplementation of l-arginine, thereby reversing the ratio of l-arginine to its methylated product, in elderly patients improved flow-mediated dilation, an indicator of endothelium-dependent vasorelaxation [51]. In older Wistar Kyoto (WKY) rats, oral supplementation with l-arginine together with an ACE (angiotensin converting enzyme) inhibitor improved blood pressure parameters; ACE is the enzyme responsible for the synthesis of Ang II (a potent vasoconstrictor) from angiotensin I [52]. Further, in the aged spontaneously hypertensive rats (SHR), l-arginine supplementation alone mitigated the effects of hypertension in the aged animals by reducing arterial pressure and reducing peripheral resistance [53].

Additionally COX-mediated alterations in eicosanoids can also be of significant consequence in an aging endothelium [46,54]. There are six eicosanoids whose synthesis is driven by COX, namely prostaglandin I_2_ (PGI_2_ or prostacyclin), prostaglandin H_2_ (PGH_2_), prostaglandin E_2_, prostaglandin F_2α_ (PGF_2α_), prostaglandin D_2_ (PGD_2_) and thromboxane A_2_ (TxA_2_). It has been widely accepted that PGE_2_, PGI_2_ and PGD_2_ are vasorelaxants, whereas TxA_2_ and the endoperoxide, PGH_2_, are vasoconstrictors [46,54]. With aging, the ratio of the vasoconstrictive factors is increased, and this plays a significant role in the age-related vascular resistance seen in elderly patients. Singh *et al.*, 2002, reported a blunted constrictor response measured in older patients compared to younger ones in the presence of aspirin (a COX-2 inhibitor) or l-NNMA (an NOS inhibitor) as measured by forearm plethysmography [55]. The activation and gene expression of COX-2 is meditated by the redox-sensitive transcription factor, NF-κB [56]. NF-κB is found to be responsible for activating inflammatory responses within the endothelial and smooth muscle cells in the vessel wall, mediating the migration of inflammatory cells into this space, as well as increasing the synthesis of proinflammatory cytokines, like IL-6 and IL-8.

The circulating levels of TNFα, IL-6, IL-1β and IL-17 are increased with aging, as seen in aged Fisher 344 rats and in a cohort of elderly subjects [57–59]. Another study on 131 males aged 81 years found a correlation between high levels of circulating TNFα and the diagnosis of atherosclerosis, with little or no association being found between TNFα and other parameters of atherosclerosis risk assessment, such as triglyceride concentration and high density lipoprotein (HDL)/total cholesterol ratio [58]. It has been suggested that increased TNFα activates NAD(P)H and leads to oxidative stress via increased ROS generation, and therefore, TNFα is a robust regulator of NF-κB [60]. A comparison of the expression of NF-κB in young *versus* old healthy humans in arterial and venous samples showed that p65, the active subunit of NF-κB, was elevated in the elderly patients; additionally, NAD(P)H was also elevated. Interestingly, the levels of xanthine oxidase and the antioxidant enzymes, MnSOD, CuSOD and catalase, were unaltered in these subjects [61]. Moreover, the NF-κB activation in the endothelium and resultant upregulation of TNFα result in increased synthesis of other chemokines, like IL-6 and synthesis of myocyte chemoattractant protein-1 (MCP-1), and adhesion factors, like intercellular cell adhesion molecule-1 (ICAM-1), which influence the migration of monocytes and the activation of T-cells, respectively. Together, they coordinate an extensive inflammatory response that is a precursor to the massive inflammation-mediated damage [62]. Furthermore, the expression of vascular cell adhesion molecule-1 (VCAM-1) is increased upon NF-κB activation in endothelial cells [63]. These alterations in the inflammatory microenvironment aggravate the inflammatory response by aiding in the binding of monocytes and activated T-cells to the vascular endothelium and triggering a variety of signal transduction mechanisms that are involved in the early phases of atherosclerosis [62,64]. Early atherosclerotic plaques consist of immune cells, endothelial cells and vascular smooth muscle cells with abundant lipid and acellular debris. These plaques, also called fatty streaks, are formed within the intima of the arterial wall initially with infiltration of more immune cells, such as macrophages. Eventually, they accumulate more lipids in a core that is surrounded by smooth muscle cells, other immune cells, like dendritic cells and T killer cells, in addition to developing a collagen-dense matrix, resulting in a mature plaque or an atheroma [64].

In a study on 18–89-year-old individuals, with a mix of healthy subjects or those with peripheral arterial occlusive disease or with coronary heart disease, the association of circulating VCAM, ICAM (cVAM-1, cICAM-1) and E-selectin, another adhesive molecule found on endothelial cells, was evaluated. The study concluded that E-selectin was not correlated with age; cICAM-1 was elevated in only atherosclerosis patients and was not dependent on age, but cVCAM-1 showed an age-dependent elevation, as it increased even in healthy subjects not having a diagnosis of atherosclerosis [65]. This study highlights a link between VCAM and biological aging and corroborates the findings made by Morisaki *et al.*, 1997, that cVCAM-1, and not cICAM-1, is correlated with age [66]. Importantly, this soluble or circulating fraction of VCAM is a resultant of the shedding of this molecule from the endothelial membrane and is a good indicator of the increase in expression of membrane-bound VCAM [67].

Matrix metalloproteinases (MMPs) are also critical in atherosclerosis progression, as these gelatinases aid in dissolving the extracellular matrix, consequently allowing more migration and infiltration of immune cells. In a study with non-human primates, monkeys, a significant increase in the MMP-2 expression and activity in the arterial wall was observed in older animals, and this was accompanied by an increase in the expression of ACE and localization of Ang II to this site [68]. Other proatherogenic molecules include CD40 and CD40 Ligand (CD40L), which are expressed on a variety of cell types, like macrophages, T-cells and platelets, and can trigger the release of proinflammatory cytokines, MMPs and other adhesion molecules, resulting in amplification of the immune response [64]. Age-related changes in platelet activation can result in hypercoagulability of the blood and result in increased thrombus formation in older adults. These changes include alterations in factors VII and VIII and fibrinogen levels and plasminogen activator inhibitor 1 (PAI-1). PAI-1 inhibits both tissue type and urokinase type plasminogen activators, which lead to fibrinolysis or inhibition of clot formation. PAI-1, an inhibitor of uPA and tPA, and, thereby, fibrinolysis, is produced in endothelial and smooth muscle cells, secreted from platelets and increases with aging [69]. Increased PAI-1 consequently leads to increased thrombus formation. Stress can also increase PAI-1 synthesis, more predominantly in aged populations, as seen in the study by Yamamato *et al.* on the effect of restraint stress on young and old mice [70]. Furthermore, as discussed previously, TxA_2_ is increased via COX-2 activation, and this is a major eicosanoid that potentiates platelet activation.

Additionally, as mentioned earlier, the inducible form of NOS, iNOS, is also upregulated in aged vessels via ROS, and this is also likely mediated by NF-κB. The induction of iNOS is induced by inflammatory stimuli, such as the presence of cytokines. iNOS is synthesized in a variety of cell types, including macrophages and epithelial cells [71]. iNOS is elevated in vessels of older animals; its inhibition using a 1400-inhibitor decreased beta-adrenergic stimulation of iNOS synthesis, related NO expression and NO-derived RNS, such as peroxynitrite, thereby attenuating the adverse effects of these RNS overall by reducing apoptosis and myocardial injury [72,73]. Senescence of vascular endothelial cells is also a contributing factor to endothelial dysfunction in aging systems, since decreased eNOS and elevated ROS and TxA_2_ have been found in these cells [46]. Cellular senescence has been attributed to telomere shortening, due to diminished telomerase reverse transcriptase (TERT) activity, and this can be regulated by eNOS, which is redox-sensitive [74]. It is accepted that oxidative stress does more damage to shortened telomeres, making cells more susceptible to senescence [75].

The transcription factors belonging to the peroxisome proliferator-activated receptor family (PPAR), regulators of cell proliferation and differentiation, amongst other cellular processes, are also redox-sensitive, with the α and the γ isotypes being involved in the regulation of inflammatory responses [71,76]. PPARs can modulate the gene expression of NF-κB and, therefore, the expression of targets downstream of this protein [77]. Thus, many of the effects seen involving PPARs are anti-inflammatory by virtue of their ability to inhibit NF-κB. For example, PPAR α-null mice demonstrated an exacerbated response to LPS. PPAR α activator was able to modulate IL-6, the downstream target of NF-κB [77]. PPARs are significantly reduced during aging. Aged Fisher rats administered 4 mg/kg of PPAR activator 2,4-thiazolidinedione (2,4-TZD) for 10 days showed reduced age-related increases in oxidative stress and NF-κB-regulated genes, such as IL-6 and IL-1β, a reduction in adhesion molecules and a reduction in COX-2 and iNOS in the kidneys of these animals [78]. In another study employing Atorvastatin (AVT, also known as Lipitor), a lipid-lowering drug, the effect of the drug on PPAR activation-driven lipid metabolism and its consequences on cardiac architecture were analyzed in 20-month-old Wistar rats. Compared to the young animals, the older rats showed left ventricular thickening, collagen deposition and reduced levels of SOD, CAT and NOS. However, with AVT treatment, the thickening of the ventricular wall was reversed, as well as significant increases in SOD, CAT and NOS activities. Further, AVT treatment inhibited the synthesis of inflammatory cytokines, TNFα, Il-1-β and IL-6, and increased the expression of PPARs in the myocardial cells isolated from these animals [79]. Thus, AVT is a good candidate for reversing age-related cardiac disorders.

Another well-established criterion for reversing the effects of aging is calorie restriction (CR). A variety of studies have laid the foundation for the potential of CR to suppress oxidative stress, be anti-inflammatory, mitigate the age-related changes in PPAR expression and its DNA binding activity, improve endothelial function and augment the expression of another critical gene linked to longevity, SIRT-1 [80–83]. SIRT-1, a member of the sirtuins family of genes, is a NAD-dependent histone deacetylase, which is involved in cell differentiation, response to nutrients and regulation of telomere length, amongst other functions [84]. CR studies in Fisher 344 rats showed that rats fed ad libitum (AL) had a debilitated response to acetylcholine-driven induction of relaxation in the aortas, increased NF-κB and vascular oxidative stress. Further, serum obtained from the CR- and AL-fed animals was tested for their antioxidant and anti-inflammatory property *in vitro* on vascular coronary endothelial cells in the presence of TNFα. It was observed that in contrast to the AL serum, CR serum did not elicit TNFα-induced ROS, and importantly, siRNA against SIRT-1 mitigated this response [83].

#### 3.2.2. Cardiac Adaptation and Molecular Basis for Malfunctioning of the Heart

Myocyte loss is a hallmark of the aging heart, resulting in replacement by sarcomeres and eventually leading to hypertrophy. Increased cardiomyocyte death debilitates optimum contractility, and the ensuing hypertrophy and remodeling can eventually cause heart failure [85]. Cardiac troponin T, a protein related with structural changes in the heart and adverse cardiovascular events, is elevated in elderly subjects [86,87]. Increased levels of troponin is an indication of myocyte injury and cell death [88]. Cardiomyocyte apoptosis is a primary cause of cell loss during aging, mediated by the BcL2/Bax pathway and a decreased PI-3-kinase/Akt pathway activation in older animals [89]. A gene profiling study investigating the gene expression alterations in older mice compared to young ones showed that genes that primarily function in stress response, mitochondrial functioning, death pathways and cytoskeletal structure are altered in the older cardiomyocytes [90]. Age-related increases in the myocyte area and increased length of relengthening (the ratio of lengthening to relengthening being a measure of myocyte contractility) have also been reported in cardiac myocytes in older mice. On closer examination, the authors found that myocytes from the aged hearts had an increased GSH/GSSG ratio, indicating oxidative stress, NAD(P)H activation, particularly the p47^phox^ subunit, and an increase in advanced glycation end products (AGEs), which are generated upon exposure to increased ROS and are correlated with cardiovascular disease and heart failure [91,92].

Autophagy functions to maintain cellular homeostasis by either clearing post-mitotic cells, perpetuating cell survival, as in nutrient deprivation, or triggering cell death upon exposure to stress, such as increased ROS. In autophagy, cellular components are targeted for destruction in the lysosome in a systematic organelle-based manner. Autophagy is regulated by autophagy-related proteins (ATG) that are, in turn, regulated by the mammalian target of Rapamycin (mTOR) [93]. Further, increased ROS results in degradation of BcL2 and, consequently, the activation of Beclin-1, another molecule that is a key player in the autophagic response. In aging, autophagy declines, thereby reducing the clearance of cellular debris from post-mitotic cells, increasing ROS and, ultimately, leading to ROS-mediated cell death [93]. Autophagy plays an important role in cardiac hypertrophy and ventricular remodeling that develops with cardiac aging, in ischemia/reperfusion (I/R) injury and acute myocardial infarction (AMI) [94]. Analyses of the left ventricle of aged mice showed that the heart mass index and the left ventricular mass index increased with age. Further, Beclin-1 and microtubule-associated protein 1, light chain 3 (LC3), were significantly reduced and BcL-2 was increased, and this was accompanied with activation of JNK1/2 and suppression of p38MAPK, leading to decreased autophagy. However, caspase3 levels remained unchanged as compared to their younger counterparts, indicating no change in apoptosis rates [95]. The occurrence of autophagy in the heart has been found to be higher than in the liver, and in older Fisher 344 rats, a reduction in ATG 7, 8 and 9 and LC3 was observed, with aging and calorie restriction (CR) reversing these effects [96]. CR has also proven beneficial in protecting against the aging heart and in protecting cardiac myocytes against apoptosis. A report on young and old rats fed a control diet (CD) or a calorie restricted (CR) diet showed that the older rats on the CR diet for six months lost body weight and showed a significant reduction in left ventricular weight compared to the old ones on the CD diet. CR enhanced mitochondrial function in isolated cardiac fibers from older rat hearts. Reduction in oxidative damage to myocytes was apparent by a decrease in 8-OHdG in the left ventricle. Reduction in apoptotic markers, like BcL-xL, caspase 3 and 9, accompanied by reduced DNA fragmentation, were observed in myocytes from CR hearts from aged animals. Intriguingly, serum obtained from these animals bestowed protective effects on H9C2 cardiomyoblast cells and cardiomyocytes from adult rats grown in culture from H_2_O_2_-mediated oxidative stress [97]. In fact, a more comprehensive analysis of CR in protecting cardiac cells from aging related damage was reported by Lee *et al.* in their gene profiling analysis studies on aging cardiac cells [98]. Their study, performed on mice, showed that CR was far more effective in altering the age-associated transcriptional pattern of genes involved in energy metabolism, extracellular matrix, structural components and apoptosis cytoskeletal changes than dietary inclusion of antioxidants, like alpha lipoic acid or coenzyme Q. However, based on the transcriptional profiles, the authors concluded that unlike reported in the skeletal muscle or in the brain, where CR acts by reducing oxidative stress and preventing oxidative damage, in the heart, it offers protection by preventing myocyte apoptosis [98–100].

Furthermore, mitochondrial dysfunction has been associated with age-related cardiac changes, with mitochondria making up for about 20%–30% of a cardiac myocyte cell volume. Furthermore, reduced mitochondrial biogenesis has been linked with the development of cardiomyopathy. Most of these mitochondrial changes have been attributed to the mutations in mtDNA due to increased oxidative stress [101]. Additionally, two distinct populations of mitochondria exist within cardiac cells, one right under the plasma membrane, called the subsarcolemmal mitochondria (SSM), and the other are the interfibrillar mitochondria (IFM) located between the myofibrils. The SSM from aged hearts remain functionally similar to the SSM from the hearts of young animals; however, the IFM are altered in that the activity of the electron transport chain is reduced [102]. The attrition of mitochondrial biogenesis can occur as a consequence of defective activation of the AMPK pathway and a significant reduction in PGC1α, a cofactor necessary for mitochondrial biogenesis [103]. PGC1α (and PGC1β) is highly expressed in the heart and is crucial in energy metabolism in this tissue, together with several transcription factors, like PPARs (discussed earlier), which are upregulated with CR in the aging heart [104]. Further, the oxidative phosphorylation (Ox-Phos) machinery could also be damaged in aging mitochondria, and disruption of Ca^2+^ homeostasis finally leads to mitochondrial permeability transition pore opening and, ultimately, activation of apoptosis. The alterations in mitochondrial function in the aging heart can predispose it to the complication of myocardial ischemia/reperfusion (I/R) injuries and to exacerbating the extent of damage [102]. In I/R, obstruction of blood flow through the coronary artery results in lack of oxygen, and when the blood flow is restored during the reperfusion phase, a vast amount of ROS is released. Oxidative damage to cells ensues and, subsequently, myocardial infarction. The damaged Ox-Phos machinery in aging is susceptible to further deterioration during I/R, accompanied by a loss of cardiolipin, a phospholipid that is exclusive to the mitochondrial membrane [105]. Moreover, lipid peroxidation of mitochondrial protein by 4-hydroxy-2-noneal (HNE), a product of oxidative damage to lipids, has been reported in hearts from older rats that have undergone I/R during the reperfusion phase [106]. Aging aggravates the injury that ensues during myocardial I/R, which was demonstrated in a study that revealed increased markers for apoptosis in the blood, such as TNFα, IL-6 and the soluble Fas ligand of elderly patients; the same result was also seen in rats after induction of I/R accompanied by increased apoptosis of myocytes evident by TUNEL staining and caspase-3 activation [107]. I/R-aged Fisher 344 rat hearts, indeed, showed an upregulation of hypertrophy- and apoptosis-related genes, as analyzed by gene expression profiling [108]. Ischemic preconditioning (IPC) is an approach that has been employed to protect the heart from injuries during I/R. Similarly, ischemic post-conditioning has been employed to protect cardiac tissue from reperfusion damage. However, the efficiency of both has been limited with age. This is likely due to a disarrayed redox balance within the mitochondria and an impaired Ox-Phos machinery, in addition to altered signaling mechanism and transcriptional regulation in an aging system [105]. Interestingly, both physical activity and CR in tandem in the elderly were able to conserve the protection offered by IPC [109].

#### 3.2.3. The Renin Angiotensin System and the Effects on an Aging Cardiovascular System

The renin-angiotensin system (RAS) is involved in blood pressure homeostasis and fluid and electrolyte equilibrium within the body. Renin, synthesized in the renal cortex, cleaves angiotensinogen to angiotensin I (Ang I), which is further acted upon by angiotensin cleaving enzyme (ACE) to form angiotensin II (Ang II) [110]. Ang II is the central effector molecule of the RAS system and is a potent vasoconstrictor molecule that mediates its effects via the G protein-coupled receptor angiotensin type 1 and type 2 (AT1 and AT2), with AT1 being expressed in the kidney, brain, heart and numerous other tissue, giving rise to a local tissue-based RAS in addition to the systemic RAS. AT1 and AT2 are antagonistic in their regulatory mechanism [110]. The RAS system is increased in sensitivity to stimuli and is overactive in an aging physiological system. An increased metabolic clearance rate accompanied by increased circulating Ang II is observed, likely because of increased synthesis as a compensatory mechanism to make up for increased clearance [68,111,112]. Moreover, Ang II is known to stimulate the production of ROS via NAD(P)H oxidase and AT1R activation and to contribute to hypertension and the onset of structural vascular changes in the blood vessels, culminating in age-related vascular complications [23,110,113,114]. Ang II is also involved in cardiac remodeling, and in this regard, ACE inhibitors, blockers of AT1R and other inhibitors of RAS activation have proven beneficial in reversing some of the alterations seen in cardiac morphology in the elderly, such as left ventricle hypertrophy, left ventricular function and congestive heart failure [115,116]. Interestingly, mice that are homozygous knockouts of *Agtr1a* (*Agtr1a*^−/−^) the gene that encodes AT_1A_, have fewer aortic atherosclerotic lesions and a lifespan that is 26% more than the wild-type counterparts. This was correlated with an increased number of mitochondria in the kidney along with upregulation of nicotinamide phosphoribosyl transferase (Nampt) and sirtuin 3 (Sirt3) in the kidney, thereby attenuating oxidative stress parameters [117]. Intriguingly, both CR and RAS blockade result in increased lifespan and, coincidentally, both result in mitochondrial protection accompanied by PPAR activation, which could be the common underlying molecular link between the two modes of increased longevity [118]. Further, the actions of Ang II and RAS extend not only to blood pressure and electrolyte homeostasis, but also to inflammation. Since Ang II can increase NAD(P)H oxidase and, thereby, the redox-sensitive transcription factor, NF-κB, the gene expression of the targets downstream of NF-κB, can, in turn, be switched on, such as MCP-1, IL-6 and adhesion factors, VCAM-1 and ICAM-1 (discussed above). Therefore, this makes Ang II a good candidate for inflammatory tissue damage and cardiac remodeling in aging [60,110,119].

#### 3.2.4. Beta-Adrenergic Responses and Effects on the Aging Cardiovascular System

The β adrenergic receptors (βAR) are G protein-coupled receptors that mediate the signaling responses to catecholamines. There are three kinds of βARs, namely, β_1_AR, β_2_AR and β_3_AR, of which β_1_AR is found primarily in the heart [120]. The sensitivity of βAR signaling decreases with increasing age with evidence of decreased βA- mediated relaxation in the elderly, likely due to decreased β_2_AR receptor density and decreased adenylyl cyclase type 5 (AC-5) activity, AC-5 being a downstream player in β adrenergic signaling. Furthermore, COX 2 and prostacyclin have also been implicated in this process [121]. Strong evidence for β adrenergic signaling being an important player in the aging of the cardiovascular system comes from studies on disruption of AC-5 being protective in age-related cardiovascular complications and an increase in longevity. AC5 knockout mice had a longer lifespan than control wild types, with an increased ability to counteract stresses, like pressure overload, stimulation by catecholamines, retarded aging phenotype accompanied by increased Raf/MEK/ERK signaling and, consequently, upregulation of SOD [122]. Chronic β adrenergic signaling can increase ROS in the mitochondria and can induce cardiomyocyte cell death. The AC5 knockout mice injected with isoproterenol, a β adrenergic agonist, showed decreased cardiomyocyte cell death and an enhanced Akt signaling [123]. ROS are important in mediating βAR signaling; MnSOD and catalase mimetic agents were able to decrease βAR-mediated release of cytochrome c, in addition to a reduced JNK-mediated mitochondrial death pathway and apoptosis of ventricular myocytes [124].

## 4. Oxidative Damage in the Nervous System

### 4.1. Changes in Architecture of the Brain and CNS—The Aging Brain

Brain regions interact to allow for higher-order cognitive functions and are often co-activated. There is significantly less-coordinated activation with aging, alluding to global loss of integrative function. Connections between the medial prefrontal cortex (mPFC), posterior cingulate (pC) and lateral parietal cortex (LP) seen in young adults is considerably reduced in aged individuals. These connections may mediate the coordinated activation in young adults, and a loss of these connections could explain disruption in coordinated activity in ageing brains. At the same time, aging brains often show additional recruitment of different portions to compensate for lost cognitive ability [125].

### 4.2. Gene Regulation

It has been demonstrated that several genes are epigenetically silenced following oxidative damage to their promoter regions, resulting in a loss of neuroplasticity and loss of memory. Enrichment of the environment or histone deacetylation, leading to activation of “lost” genes, has been shown to correlate with recovered memory and better neurological function [125]. Postmitotic cells, such as neurons, have higher accumulated proteins, due to mutations, gene duplication and free radical protein damage, in addition to lower levels of intracellular protein recycling systems. The downstream effect of all of these changes is neuronal death [30,31]. A decrease in signaling pathways, such as the IGF1/Insulin pathway and TOR signaling, is correlated with the development and progression of Alzheimer’s disease. A decrease in mitochondrial efficiency and function is associated with Alzheimer’s disease, Parkinson’s disease, Cerebral Ischemic reperfusion injuries and an exacerbation of symptoms associated with Huntington’s disease [125,126]. In general, most genes in primate brains are downregulated with age in comparison with global gene expression in other tissues or in comparison with global gene expression in brains of other mammals. More specifically, genes associated with neuronal plasticity, mitochondrial function and turnover, the ubiquitin-proteasome pathway and inhibitory interneuron function are all downregulated, while genes associated with stress response, inflammation, metal ion homeostasis, myelin-related protein and glial genes are all upregulated in humans [125].

### 4.3. Oxidative Stress and Damage in the Brain

The brain and neurological system are amongst the greatest accumulators of oxidative damage with age [6]. Some of the primary reasons are a consequence of greater oxygen consumption, a larger number of mitochondria, abundant nitric oxide, polyunsaturated fatty acid (PUFA) rich cell membranes, which are readily peroxidated, and, finally, lower levels of antioxidant enzymes, such as glutathione peroxidase (GPx) and catalase [126,127]. Oxidative damage has been linked to Alzheimer’s disease (AD), Parkinson’s disease (PD), Amyloid Lateral Sclerosis (AML), Multiple Sclerosis (MS), mild cognitive impairment (MCI), Huntington’s disease, cerebral ischemia and HIV-associated neurocognitive disorder (HAND). Progression of disease has been attributed to several factors, some of which are reduced levels of exogenous and endogenous antioxidants, increased oxygen consumption in the brain, higher numbers of mitochondria in neurons and increased levels of nitric oxide. A greater number of damaged mitochondria are also seen in the case of AD, PD and MS [126–128]. For the purposes of this review, discussion is limited to oxidative damage associated with AD, cerebrovascular events, PD, MCI and Huntington’s disease, all of which are selectively higher in older adults. In statistical terms, free radical damage contributes to a 50% probability that an older adult over 85 will have Alzheimer’s disease. The primary cause of age-related neurological decline has been attributed to oxidative stress [125]. PD is the second most prevalent neurodegenerative brain disorder, affecting up to 2% of the population over 65 years of age [129].

### 4.4. Molecular Markers of Neuronal Oxidative Damage

To study neurological damage, it is essential that several different markers be measured to ascertain oxidative damage. In a recent study, leukocyte 8-OHdG and plasma malondialdehyde (MDA), as well as antioxidants, erythrocyte GPx activities and plasma vitamin E levels were used to correlate the severity of Parkinson’s Disease (PD) with measures of oxidative damage [130]. Assays for oxidative damage to nucleic acids are determined in several different ways. Levels of 8 oxodG are determined enzymatically, followed by single cell gel electrophoresis (COMET) or by using mass spectroscopy and ELISA. Other measures of oxidant damage to DNA, such as single-stranded breaks are also decipherable using the COMET assay [3]. Double-stranded breaks are visualized as gamma-H2AX (g-H2AX) foci, which can be detected immunologically [131]. Levels of the antioxidant, glutathione (GSH), in erythrocytes are also an accepted measure of oxidative damage. GSH levels in RBCs of AD patients is lower than levels seen in adults with normative mental aging [25,132]. Oxidation of lipids can be reliably measured using F2 isoprostanes, 4-hydroxy-2,3-trans-nonenal (HNE) or malondialdehyde as markers of lipid peroxidation [132,133].

### 4.5. Alzheimer’s Disease

Alzheimer’s disease (AD) is characterized by a loss of neurons in the hippocampus and associative cortex. AD occurs primarily because of associated autosomally dominant genes on chromosome 21, and on occasion, its occurrence is sporadic. At the organ level, there is non-normative decrease in the regional medial temporal, lateral temporal and parietal lobes of the brain. Furthermore, there is a decrease in deep frontal white matter, the genu of the corpus callosum and atrophy in the hippocampus [134]. Accumulation of the amyloid β protein (Aβ) extracellularly in senile plaques, loss of dendrites and neural spines and neurofibrillary tangles are seen at the cellular and subcellular levels [135]. Subsequent dysfunction in a second protein, tau, a microtubule-associated protein, results in dismantling of the microtubules in the cell. Neuro fibrillary tangles are a result of the disassembly of tau, as a result of its hyperphosphorylation [136]. Most gross visualization of the brain in AD is by MRI, PET or by microscopy of brain tissue [137]. At a molecular level, mitochondrial DNA of Alzheimer’s patients shows more mutations, an accumulation of Aβ in mitochondria and in certain areas of the brain, leading to inflammation and increased RONS production at those sites. Additionally, there is an absence of cytochrome complex IV in hippocampal neurons, which adds to greater mitochondrial dysfunction and greater oxidative stress [135,138]. The cytopathology and progression of AD is explained by two competing hypotheses, the first of which suggests an amyloid-driven process, and the second suggests a dysfunctional mitochondria-driven process [135]. The amyloid cascade hypothesis owes its origin to Glenner and Wong, who discovered a 4 kDa peptide that they labeled Aβ protein [139]. The hypothesis has evolved over the past 25 years to basically suggest that deposition of Aβ in brain parenchyma as a downstream result of aberrant cleavage of its precursor protein, APP, either due to mutations in APP or due to mutations in AD-associated presenilin genes, PSEN1 and 2, leads to neurofibrillary tangles via tau and, ultimately, neuronal death [140]. This occurs via the entry of Aβ into the mitochondria and its binding to mitochondrial alcohol dehydrogenase, resulting in the release of cytochrome c and, consequently, causing apoptosis [138]. This classical hypothesis considers AD and brain aging to be uniformly divergent, which is contentious according to the proponents of the mitochondrial cascade hypothesis proponents. It also considers most oxidative damage in AD to be amyloid-driven. According to the proponents of the mitochondrial cascade, progression of AD in sporadic occurrences are driven by aging-associated mitochondrial dysfunction, which leads to hyperphosphorylation of tau and, thereby, the neuron’s increased sensitivity to Aβ [141].

### 4.6. Ischemic Stroke

Cerebrovascular events, such as ischemic strokes (IS) as a consequence of atherosclerosis (described earlier), play a significant role in the progression of AD. In and of themselves, IS and subsequent reperfusion result in considerable oxidative damage, leading to neuronal death [126]. Furthermore, cerebral ischemia is correlated with an increase in Aβ in neurons. Aβ accumulation, in turn, could increase inflammatory events along the blood brain barrier (BBB), causing cerebrovascular dysfunction. Triggering of proinflammatory genes via TNFα, such as IL-1β, IL-6 and iNOS, increases oxidative stress and the potential for apoptosis in neurons [142]. A significant amount of damage occurs at the interface of the circulatory system and the central nervous system, a barrier well known as the blood brain barrier (BBB), in several neurological disorders as a result of oxidative damage at the blood brain barrier. Oxidative damage in blood vessels at the BBB causes immune cells to aggregate at the site and infiltrate into the brain. This infiltration is accompanied by inflammation and complex activation of all types of neural cells, resulting, ultimately, in neuronal death [128,143].

### 4.7. Parkinson’s Disease

In the case of Parkinson’s disease (PD), mitochondrial defects in complex I and an increased iron content in the substantia nigra and frontal cortex result in selective loss of dopaminergic neurons. The oxidation of dopamine is considered a driving force for the development of PD. It is believed that the process of oxidation of dopamine can occur enzymatically or spontaneously [144]. Dopamine, when oxidized, is converted to neuromelanine. Neuromelanine, along with ferritin, primarily functions to sequester extra iron in dopaminergic neurons. An excess of iron, which catalyzes the production of the OH• radical from hydrogen peroxide results in lipid peroxidation and cellular damage [129]. The oxidative status of PD brains is often reflected in components of peripheral tissues, such as blood. This is significant from a therapeutic perspective, as it enables measurement of disease progression following therapy [130]. MPTP (1-methyl-4-phenyl-1,2,3,6-tetrahydropyridine) is a neurotoxin that has been used to study PD and its progression in animal models. MPTP is metabolized to MPP^+^ (1-methyl-4-phenyl pyridinium), which is taken up by mitochondria of dopaminergic neurons of the substantia nigra (SN). MPP^+^ then binds to complex I of the electron transport chain, inhibiting it and, thereby, generating ROS, which ultimately leads to cell death [145]. Deletions and mutations in mitochondrial DNA (mtDNA) have been shown to play a role in the heredity of PD. Other genes that form the basis of the heredity of PD are the mitochondrial serine/threonine kinase, PINK1, the E3 ubiquitin ligase, Parkin, the redox-regulated chaperone, DJ-1, leucine-rich repeat kinase 2, (LRRK2) and the presynaptic protein, α-synuclein. These genes have been shown to be associated with oxidative protection or are mitochondrial in function [146]. PINK1 is thought to phosphorylate mitochondrial proteins in response to oxidative stress, primarily as a neuroprotective function. Mutations in PINK1 are associated with autosomally recessive PD. PINK1 also signals and activates intracellular Parkin to ubiquitinate defective mitochondria for mitophagy. In the absence of this key mechanism for mitochondrial turnover, defective mitochondria accumulate in the neuron, resulting in increased oxidative stress and damage [129]. Protein accumulation in PD occurs, much like AD, and the protein that accumulates is α synuclein. These are visualized as Lewy bodies. α synuclein is found presynaptically and has been proposed to play a role in presynaptic signaling and neuroplasticity. Additionally, α synuclein is believed to regulate mitochondrial membrane lipid composition and complex I activity. Over-production of α synuclein and its post-translational modification as a consequence of oxidative stress result in permeabilization of the mitochondrial membrane by α synuclein and a loss of membrane potential. This is accompanied by the release of cytochrome c and cell death [129,146]. Accumulation of α synuclein presynaptically promotes mitochondrial dysfunction and an increase in RONS [127].

### 4.8. Mild Cognitive Impairment

Mild cognitive impairment (MCI) is altered cognitive and memory function, which is not severe enough to be classified as dementia. Levels of apolipoprotein E (APOE) are increased in MCI patients who later develop AD. Markers of oxidative damage, evidenced as thiobarbituric acid-reactive substances (TBARS), malondialdehyde (MDA), HNE and acrolein are elevated in the superior and middle temporal gyri in patients with MCI. Levels of F2-isoprostanes in different brain regions were also seen to be elevated. On the basis of oxidative stress reporter molecules, MCI mimics AD [147]. Overall brain volumes in MCI are not reduced as dramatically as in AD when comparing the amygdala and hippocampus. Changes in regional cerebral blood flow (rCBF) were mostly insignificant for most brain regions and unrelated to brain volumes in MCI [148]. It is considered to be a precursor to dementia, particularly, Alzheimer’s disease [149–151]. Several physiological markers, such as increased oxidative damage of DNA in peripheral leucocytes, higher levels of lipid peroxidation, lower levels of both enzymatic and non-enzymatic anti-oxidants and damage to aconitase, a key enzyme in the citric acid cycle, contribute to the increased oxidative stress that cause the progression of MCI to dementia [126].

### 4.9. Huntington’s Disease

Huntington’s disease (HD) is a hereditary neurodegenerative disorder that starts in the fourth and fifth decade of life and develops over the next 10–15 years. The pathology of the disease is due to site-specific mutations of the huntingtin gene (Htt) in its first exon that lead to CAG repeats. The function of the 350 kDa protein is unclear, and it might have a role in apoptosis inhibition and vesicle trafficking. The mutant (mHtt), with its internal polyglutamine stretch, results in neurodegeneration. There is significant atrophy of neurons in the caudate and putamen prior to clinical symptoms becoming apparent. Oxidative damage dramatically worsens the physiological impact of HD. HD patients often show impairment in complex II and III of the oxidative phosphorylation pathway. All the classic markers of oxidative damage are seen in increased quantities, such as 8-OHdG, F2-isoprostanes and protein carbonyls. In particular, proteins involved in ATP generation appear to be impacted [126,152–154].

## 5. Caloric Restriction and Damage Mitigation

Caloric restriction (CR) with adequate micronutrient supplementation leads to an increase in life expectancy in laboratory animals. By far, there is a consensus on the ability of CR to reduce oxidative damage in several types of tissues, particularly of the cardiovascular and nervous systems, thereby increasing life expectancy [155–161]. Studies suggest several synergistic mechanisms that work in tandem to enhance life span. Increase in NO with a combination of reduced ROS is both neuro and cardioprotective, due to activation of the Nrf2 antioxidant pathway. This activation also is anti-inflammatory [162]. There is also a potential role for the sirtuins (SIRT1, 3, 4, 5) involvement in reducing the impact of oxidative damage in post-translationally modified mitochondrial proteins, although the mechanism is unclear [163]. SIRT1 in particular, has been shown to reduce accumulation of Aβ in AD [164]. Interestingly, a recent study has found that reduction of protein intake and, more specifically, methionine replacement in diet by 80% also has the potential to increase life expectancy in rats. The research group found a significant reduction in ROS and oxidative damage [160]. This correlates well to the oxidative damage in brains of laboratory animals associated with oxidation of methionine in its proteins. Locomotor activity and brain dopamine levels of methionine sulfoxide reductase knockout (Msr^−/−^) mice are considerably altered, unless the Msr^−/−^ are on caloric restriction [159]. From a therapeutic standpoint, the focus on caloric restriction in neural aging has resulted in the identification of several potential targets, such as the sirtuins, BDNF, FoxO and PPAR [158].

Within the realm of cardiovascular disorders, CR reduces oxidative stress-induced induction of proinflammatory markers, like NF-κB-mediated cytokine synthesis, protection from endothelial damage and reversal of the progression to atherosclerosis [82,83]. Further, a reduction in myocyte size, and cell death via apoptosis is observed in calorie-restricted aged hearts, a mechanism attributed to the protection of mitochondria from membrane collapse [97]. As discussed in section III B, these changes seen due to CR can be attributed to SIRT1 and PPAR, both central to the beneficial effects of CR seen in cardiovascular disorders. In this regard, resveratrol and other SIRT activators have been identified as potential compounds that can increase longevity and improve cardiac health [71,80,84,85]. Further, RAS inhibition and CR seem to have converging effects in their mechanism, both mediated by PPAR upregulation [118].

## 6. Summary Remarks

Reactive chemical species, produced endogenously by the mitochondria and exogenously by exposure to air pollution or UV light, amongst other causes, in excess impacts a variety of cell types and their functionalities. During aging, oxidative damage is accelerated, likely due to decreased antioxidant capacity and altered signaling mechanisms, rendering tissues and organs susceptible to damage. The impact on physical and mental health along with life expectancy is considerable. Numerous strategies have been suggested to reduce the burden of oxidative stress on the aging body. Dietary intervention studies with antioxidants have shown promising results. More recently, the natural antioxidants found in plants, fruits and vegetables, namely polyphenols in the form of nutraceuticals, are gaining popularity, but work still remains to be done to optimize the dosage and bioavailability criteria of these products. Lifestyle changes, like moderate physical exercise, smoking cessation and intervention in sleep disorders, are known to mitigate oxidative damage in older adults. The most promising data comes from calorie restriction, providing strong evidence for reduction in oxidative stress burden and increasing longevity. Further studies that elucidate various pathways that feed in and out of cellular signaling associated with calorific restriction will help identify targets for therapeutic intervention and perhaps provide the silver bullet to inhibit or even reverse some of the impacts of oxidative damage.
